# Effect of Metformin on the Functional and Electrophysiological Recovery of Crush Injury-Induced Facial Nerve Paralysis in Diabetic Rats

**DOI:** 10.3390/jpm13091317

**Published:** 2023-08-27

**Authors:** Kyung Hoon Sun, Cheol Hee Choi, Gwang-Won Cho, Chul Ho Jang

**Affiliations:** 1 Department of Emergency Medicine, College of Medicine, Chosun University, Gwangju 61452, Republic of Korea; skhkorea@chosun.ac.kr; 2 Department of Pharmacology, College of Medicine, Chosun University, Gwangju 61452, Republic of Korea; chchoi@chosun.ac.kr; 3 Department of Biology, College of Natural Science, Chosun University, Gwangju 61452, Republic of Korea; gwcho@chosun.ac.kr; 4 BK21 FOUR Education Research Group for Age-Associated Disorder Control Technology, Department of Integrative Biological Science, Chosun University, Gwangju 61452, Republic of Korea; 5 Department of Otolaryngology, Chonnam University Medical School, Gwangju 61469, Republic of Korea; 6 Department of Otolaryngology, Gwangju Veterans Hospital, Gwangju 62284, Republic of Korea

**Keywords:** facial nerve, crush, diabetes, metformin

## Abstract

The impact of metformin on the rat facial nerve following crush injury has only occasionally been documented to date. The purpose of the current investigation was to use functional and electrophysiological evaluations to investigate the effects of metformin administration on recovery following crush injury to the rat facial nerve. The rats were randomly divided into four groups: the nonDM/PBS group (n = 4), the nonDM/metformin group (n = 4), the DM/PBS group (n = 4), and the DM/metformin group (n = 4). Diabetes was generated by an intraperitoneal injection of streptozotocin. Facial nerve paralysis was induced by a crush injury 7 days after diabetes induction. The blood glucose levels of the DM/PBS and DM/metformin groups were maintained at over 300 mg/dL, whereas the blood glucose levels of the nonDM/PBS and nonDM/metformin groups were maintained at less than 150 mg/dL. There was no significant difference between the two nonDM groups. In comparison to the PBS group, the metformin group’s recurrence of vibrissa fibrillation occurred noticeably sooner over time. The nonDM/metformin group showed the highest recovery rate in the second, third, and fourth weeks post-crush, respectively. The threshold of action potential 4 weeks after crush injury showed that the nonDM/metformin group had a significantly lower mean threshold of MAP compared to other groups. The short-term effect of metformin on the recovery of facial nerve blood flow (FNBF) was significantly increased compared to the DM/PBS group. However, there was no significant difference in FNBF between the nonDM/metformin and nonDM/PBS groups. A diabetic condition promoted a delay in FN regeneration. Metformin is able to accelerate functional recovery in diabetic or nondiabetic FN-injured rats. Further studies using a morphometric or molecular approach are planned to understand the pharmacologic mechanism of metformin.

## 1. Introduction

All facial nerve-innervated structures are paralyzed in a condition known as facial nerve palsy, which prevents facial expressiveness, and 10–23% of the causes of facial nerve paralysis are related to trauma, whether unintentional or brought on by tumor involvement, which can include facial paralysis [1]. The quality of life of patients is significantly impacted by facial nerve palsy, which is important.

Patients with diabetes mellitus frequently develop peripheral neuropathy, a group of clinical disorders that affect the motor, sensory, and autonomic nerves [2]. 

Several treatment strategies have been created in the last ten years to promote nerve regeneration, including topical nerve growth factors [3,4,5,6] or extracellular matrix molecules [7,8,9,10], as well as the use of electrical stimulation [11,12,13,14]. All of these techniques have been performed in animal research and, sadly, have a limited range because they have not been clinically applied, except electrical stimulation. 

Treatment of facial nerve injury is becoming more challenging due to the increased frequency of chronic diseases like diabetes mellitus (DM), and only a small number of studies have shown therapies effective for peripheral nerve regeneration in diabetes. Considering that type 2 diabetes (T2D) is so common, has a growing scope of usage, and has a positive safety profile, metformin has emerged as one of the antidiabetic medications that doctors most regularly recommend to their patients globally. Poor results may be connected to the peripheral nerve injury population’s steadily increasing prevalence of chronic diseases, such as diabetes mellitus (DM), which is anticipated to afflict 591.9 million people by 2035 [15]. Due to its direct effects on nervous system function, including axonal atrophy, segmental demyelination, and the slowly regenerating nature of injured nerves, long-term hyperglycemia, a typical manifestation of DM, considerably complicates treatment outcomes [16]. The main mechanism of action of metformin is inhibition of hepatic glucose-6-phophatase activity, which activates glycogen sparing [17]. And then it enhances the effect of insulin on glucose transport at sites beyond insulin receptor binding and phosphorylation without changing insulin receptor number or their affinity in adipose tissue. Despite the fact that metformin is the preferred treatment for type 2 diabetes, a detailed mechanism is not known about its therapeutic ability to avert or postpone peripheral nerve damage in the disease. Metformin may have a variety of effects [18,19,20], but this study could not totally rule out the benefit of the medication’s ability to lower blood sugar levels. Therefore, more investigation is needed to ascertain the mechanism of neuroprotective pathways unrelated to glucose regulation. Recently, reports have begun to emerge that metformin exhibits neuroprotective effects by suppressing oxidative stress [21,22,23]. In addition to the antioxidant effect of metformin, through AMPK and autophagy activation, metformin can improve neuronal bioenergetics, encourage nerve healing, and lessen harmful protein aggregation in neurological disorders [24]. After a peripheral nerve injury, numerous biological processes, such as inflammation, oxidative stress, hypoxia, etc., take place at the injury site. Metformin may prevent hypoxia-induced apoptosis and assist Schwann cells (SC)s in recovering from hypoxia-induced damage [25]. Metformin may also partially counteract the negative effects of hypoxia on cell proliferation, viability, migration, and adhesion [25].

To date, the effect of metformin on the sciatic nerve has been reported in a few studies [26,27,28]. We believe that metformin would be considerably more effective in the regeneration of the crush-injured facial nerve in diabetes mellitus. The impact of metformin on the rat facial nerve following a crush injury has not been documented to date. The aim of this study was to use functional and electrophysiological evaluations to investigate the effects of metformin administration on recovery following crush injury to the rat facial nerve.

## 2. Materials and Methods

### 2.1. Diabetes Induction

This study was approved by the Animal Experimentation Committee (CIACUC2021-S0021). 

Adult male Sprague Dawley rats (200–250 g, 6–8 weeks old; SamtakoBio Korea, Suwon, Republic of Korea) were used in this study. Rats were randomly divided into four groups: nonDM/PBS group (n = 4), nonDM/metformin group (n = 4), DM/PBS group (n = 4), and DM/metformin group (n = 4). Diabetes was induced by an intraperitoneal injection of streptozotocin (75 mg/kg) (Merck Korea, Seoul, Republic of Korea) dissolved in 0.1 mol/L sodium citrate buffer (30 mg/kg body weight). Phosphate-buffered saline (PBS) and metformin (100 mg/kg) were administered daily by the oral route using a flexible oral zondae needle (Jeung Do Bio & Plant Co, Seoul, Republic of Korea). Facial nerve paralysis was induced by a crush injury 7 days after diabetes induction. Using the Precision Xtra Plus instrument, blood samples were collected from the tail vein for analyzing blood glucose levels (G 400 Green Doctor, Blood Glucose Monitoring System, GCMS, Yongin City, Gyeonggi, Republic of Korea). In this study, diabetes was defined as having blood glucose levels of more than 250 mg/dL.

### 2.2. Induction of Facial Nerve Paralysis by a Crush Injury

After shaving around the left auricle, a postauricular incision was made. We preferred the left side because it allows easy setup for measuring action potential thresholds. The subcutaneous layer was dissected, and the main trunk of the facial nerve was identified after peeling the surrounding tissue under a surgical microscope (Leica, Wetzlar, Germany). A hemostat was used to crush the main trunk for one minute. This method of crushing caused damage to all nerve fibers while sparing the axonal sheath. Each rat was kept in a separate cage with free access to food and water. One week before the surgery, the animals were provided with the opportunity to settle in without stress. 

### 2.3. Assessment of Recovery of Vibrissa Movement Using Slow Video Analysis Software

The rat’s body was fixed in a modified plastic bottle while the head was freely exposed. The vibrissa movement on the left side in both groups was recorded using an iPhone video recording system after tactile stimulation using a brush 1, 2, and 3 weeks after crush injury-induced facial nerve paralysis. The number of vibrissa fibrillations was counted in slow motion using behavioral observation research interactive software (BORIS), which is used for animal behavior evaluation. This software was developed by Oliver Friard and Marco Gamba (Department of Life Sciences and Systems Biology, University of Turin, Turin, Italy) and is provided freely for research purposes. The frequency of vibrissa movement (left side vs. right normal side) was compared each week between the control and study groups. 

### 2.4. Measurement of Electrically Evoked Action Potential

The facial nerves were re-exposed under general anesthesia using isoflurane inhalation at postoperative week 4. The distal part of the crush site was stimulated using a monopolar tungsten probe, and the threshold of the action potential was measured as described previously [29]. Briefly, the midway point of the left orbicularis oculi and orbicularis oris muscles was percutaneously fixed with three two-needle electrodes. As a ground needle, it was fixed in the superficial muscle layer near the skin to record electrically evoked muscle action potential (MAP) signals. A monopolar stimulating electrode (Xomed-Treace, Jacksonville, FL, USA) attached to a pulse generator was used to send electrical impulses (rectangular current pulses for 0.05 ms) to the main trunk of the facial nerve (A-320D; World Precision Instruments Inc., Sarasota, FL, USA). A micromanipulator was used to adjust the position and direction of the monopolar stimulating probe with respect to the facial nerve. With maximum nerve stimulation, the MAP signals were assessed. A Samsung computer monitor and the lab chart system (PowerLab; AD Instrument, Castle Hill, Australia) were used to automatically collect the data, which were subsequently evaluated using the Scope software (AD Instrument). To measure the extent of recovery following a facial nerve injury, the peak amplitude of the action potential waveform was measured.

### 2.5. Nerve Blood Flow Measurement Using a Laser Doppler Blood Flowmeter

Two rats from each group at postoperative week 4 were anesthetized using an intraperitoneal injection of xylazine hydrochloride and tiletamine-zolazepam (Zoletil, Virbac, Carros, France). The recombinant position is more convenient for the measurement of FNBF when using Zoletil with xylazine hydrochloride. The main trunk of the facial nerve was carefully re-exposed, and the femoral artery was also identified. FNBF in the region was assessed using a laser Doppler blood flowmeter, as described previously [30]. The femoral artery, which is routinely used to measure systemic blood pressure (SBP), was cannulated, and a pressure transducer was attached (AD Instruments, Castle Hill, Sydney, NSW, Australia). On the main trunk of the FN, a 1.0 mm needle probe was positioned at a straight angle carefully to avoid nerve compression and coupled to a laser Doppler blood flowmeter (moorLAB, Moor Instruments, Axminster, Devon, UK). Every 20 s, data on the FNBF output and SBP were sampled and evaluated using a data acquisition program (PowerLab, AD Instruments) and a laptop (Samsung, Suwon, Republic of Korea). The FNBF was recorded for 30 min. 

### 2.6. Statistical Analysis

All statistical analyses were performed using the GraphPad Prism 8.0 software. Comparisons between the three groups were performed using a one-way ANOVA. A *p*-value less than 0.05 was considered statistically significant.

## 3. Results

### 3.1. Induction of Diabetes and Facial Nerve Paralysis by a Crush Injury

**Figure 1:**
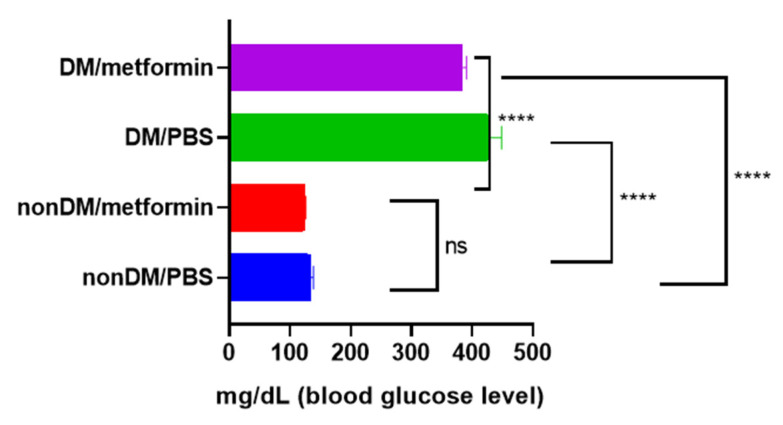
Blood glucose levels. **** *p* < 0.0001, ns: not significance.

**Figure 2:**
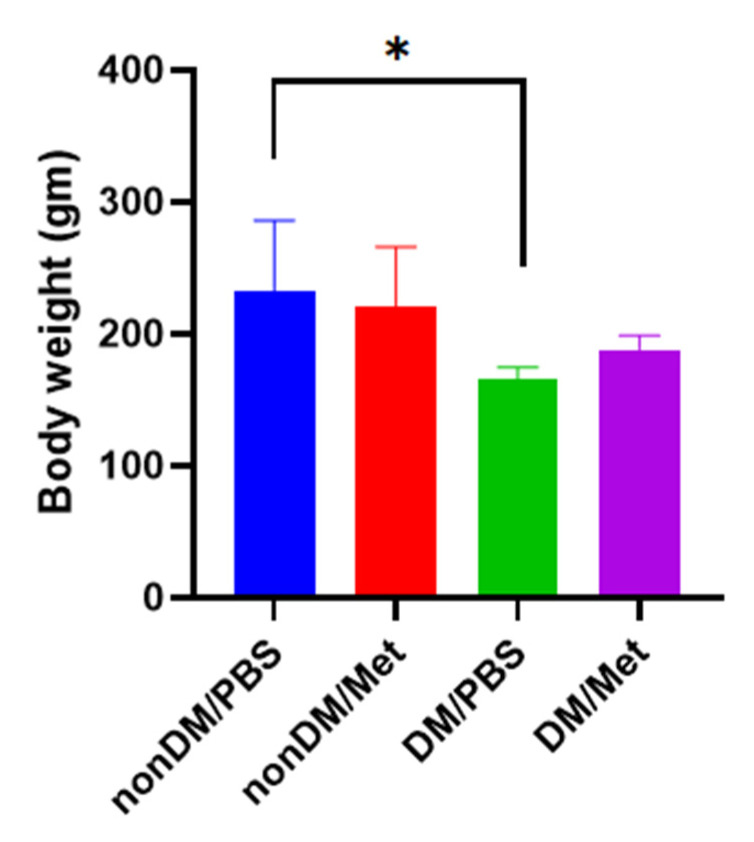
Body weight gain. * *p* < 0.05.

There were no intraoperative complications, and all rats survived after the surgery. The blood sugar levels of the diabetic group were measured daily using a portable glucose monitoring machine. The diabetic groups (DM/PBS and DM/Metformin) displayed higher glycemic levels and lower body weights than the non-diabetic groups during the entire trial period. The blood glucose levels of the DM/PBS and DM/metformin groups were maintained at over 300 mg/dL, whereas those of the nonDM/PBS and nonDM/metformin groups were maintained at less than 150 mg/dL (Figure 1). One-way ANOVA showed significant differences between the four groups (*p* < 0.0001). Furthermore, multiple comparisons by Tukey’s test showed significant differences between groups except nonDM/PBS vs. nonDM/metformin, *p* = 0.5104 (nonDM/PBS vs. DM/PBS, *p* < 0.0001; nonDM/PBS vs. DM/metformin, *p* < 0001; nonDM/metformin vs. DM/PBS, *p* < 0.0001; nonDM/metformin vs. DM/metformin, *p* < 0001; and DM/PBS vs. DM/metformin *p* = 0.0002). Body weight gain was observed in the nonDM/PBS group, but no change was observed in the DM/PBS or DM/metformin groups. One-way analysis of variance (ANOVA) showed significant differences among the four groups (*p* = 0.0358). However, post hoc Tukey’s test showed that significant differences exist only between the nonDM/PBS and DM/PBS groups (*p* = 0.0427). No differences were observed between the nonDM/PBS and nonDM/metformin groups (*p* = 0.2539) or between the DM/PBS and DM/metformin groups (*p* = 0.7458) (Figure 2). 

### 3.2. Recovery of Vibrissa Fibrillation 

**Figure 3:**
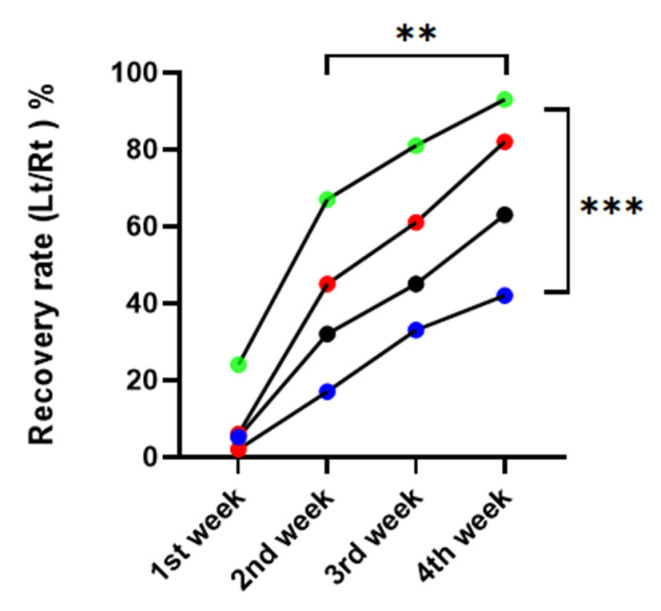
Recovery rate of vibrissa fibrillation. red: nonDM/PBSI, blue: DM/PBS, black: DM/metformin, green: nonDM/metformin (Repeated one-way ANNOVA between groups, *p* = 0.0015, between weeks, *p* = 0.0008, ** *p* < 0.01, *** *p* < 0.001.

Compared to the DM/PBS group, vibrissa fibrillation occurred noticeably sooner over time in the nonDM/metformin group. There was a significant difference between the four groups in the repeated one-way ANOVA, *p* = 0.0015. There was a significant difference between postoperative weeks, with *p* = 0.0008 (Figure 3). 

### 3.3. Recovery of the Action Potential of Facial Muscles

**Figure 4:**
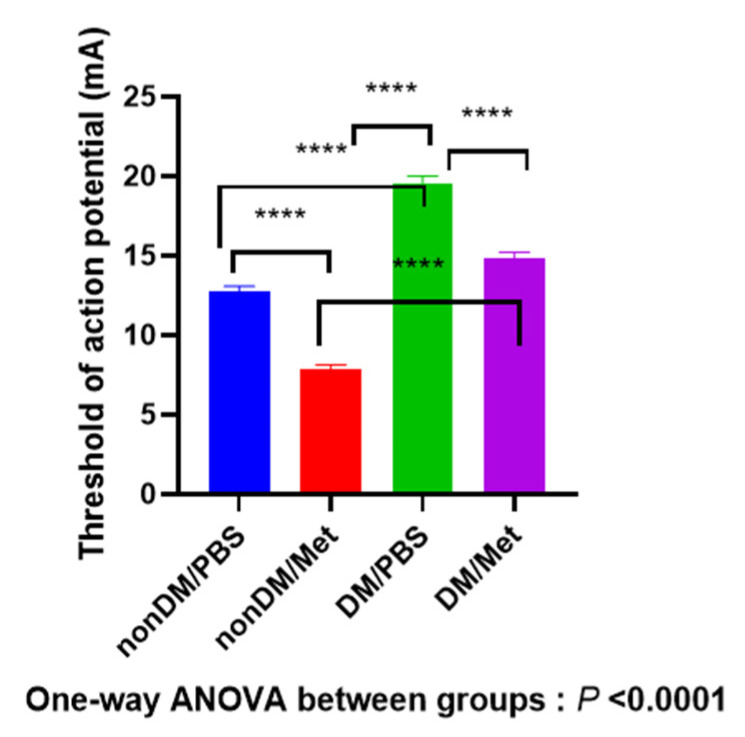
Recovery of the threshold of action potential. **** *p* < 0.0001.

As shown in Figure 4, four weeks after the crush injury, there was a significant difference between four groups (one-way ANOVA, *p* < 0.0001; multiple comparisons by Tukey’s test, nonDM/PBS vs. DM/metformin, *p* < 0.0001; nonDM/PBS vs. DM/PBS, *p* < 0.0001; nonDM/metformin vs. DM/PBS, *p* < 0.0001; nonDM/metformin vs. DM/metformin, *p* < 0.0001; DM/PBS vs. DM/metformin, *p* < 0.0001). 

### 3.4. Recovery of Facial Nerve Blood Flow

**Figure 5:**
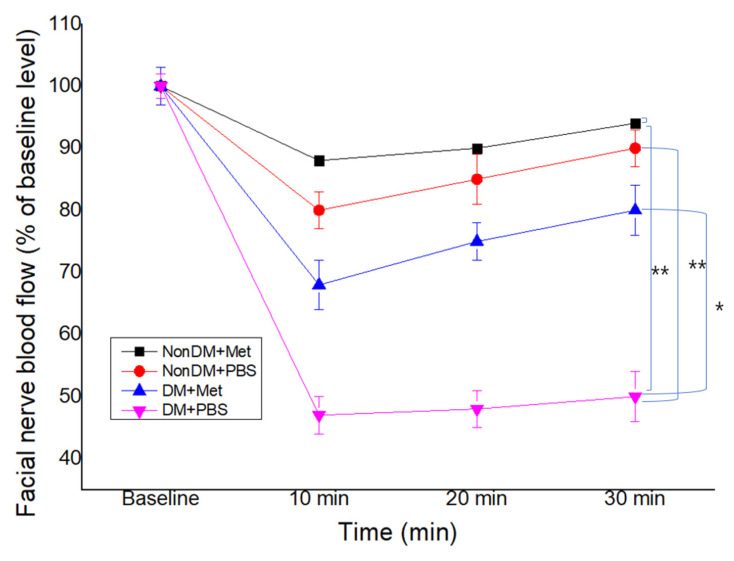
Facial nerve blood flow at 4 weeks post-crush. * *p* < 0.05, ** p < 0.01.

Compared with the nonDM/PBS group, both DM groups showed a decrease in FNBF (Sham PBS vs. DM/PBS, *p* < 0.01). The recovery of FNBF in the DM/metformin group was significantly higher than that of the DM/PBS group (Figure 5). The nonDM/metformin group showed the highest recovery of FNBF compared to other groups. 

## 4. Discussion

Axonotmesis, a condition frequently associated with crush injuries, results in significant sensory dysfunction and functional limitations [31]. As observed in our work, crush injuries resulted in a brief but total loss of function in non-diabetic rats, which returned to normal levels after 4 weeks. In the present study, we observed the effect of metformin on electrophysiological recovery. In fact, our observation period was 4 weeks. In the current study, we used animal behavior analysis software (BORIS) with video analysis to evaluate the recovery of vibrissa fibrillation. In contrast to subjective observation, this procedure was objective. Subjective observation is not a good enough tool to examine the recovery of vibrissa fibrillation in a facial nerve paralysis model. Prior to this, we made subjective observations using a modified version of Gilad’s arbitrary score [32]: 0, complete paralysis with vibrissae flattened and oriented posteriorly; 1, slight quivering vibrissae movements; moderate quivering vibrissae movements; 3, quivering movements but abnormal orientation; 4, apparently normal movements but still abnormal orientation of caudal vibrissae; 5, full movement and normal orientation. However, two or three observers are needed for this subjective evaluation. 

The severity of diabetic patients’ neuropathy is significantly connected with their glycemic management, with hyperglycemia serving as the primary causative factor. 

When compared to crush-injured non-diabetic rats, diabetic rats in our study displayed significantly lower recovery of vibrissa fibrillation values in the first three weeks following a facial nerve crush. Furthermore, while diabetes rats did not regain their motor function until post-injury week four, non-diabetic rats did so by post-injury week four. According to these results, spontaneous functional motor recovery is slower when there is persistent hyperglycemia, which may be related to problems with nerve regeneration following damage. 

It has been demonstrated that metformin is beneficial for conditions associated with diabetes, including cancer, inflammation, and heart failure. Human bone metabolism and metformin have both been studied [33]. The modest axonal regenerating rate of mammalian peripheral nervous system neurons severely restricts their ability to regenerate following injury [34]. Endoneurial ischemia, or hypoxia, is caused by a pathological alteration in the endoneurial microvessels in the peripheral nerves as a result of a crush injury [35]. This change affects both the blood flow to the nerves and the oxygen tension in the endoneurium. By producing free radicals, crushing causes oxidative stress by introducing factors such as lipid peroxidants into the neurovascular cells [36]. Instead of neuroinflammation and edema, the repair process after a nerve injury is decreased primarily by free oxygen radicals [37]. Antioxidant substances aid in the regeneration of nerves by scavenging free oxygen radicals. Mammalian species have antioxidant enzymes like superoxide dismutase and catalase, whose function is to shield the cells from the harmful effects of free radicals. A fundamental mechanism for cell death is free radical-induced traumatic cell injury. In the present study, we did not perform molecular studies for the antioxidant effect of metformin on the repair process of crush-injured facial nerves. However, the enhanced electrophysiological recovery by metformin suggests the therapeutic antioxidant effect of metformin. In the present study, the DM/metformin group showed that facial nerve blood flow was significantly better than that of the DM/PBS group. In acute streptozotocin-induced diabetes, nerve blood flow is modestly decreased, and antioxidants can improve nerve blood flow [38,39,40]. Moreover, by reducing nerve blood flow, crush damage hastens nerve ischemia. Reactive oxygen species (ROS) are produced, and hypoxia is induced as a result of this lowered nerve blood flow [30,38,41]. Metformin affects insulin resistance in these subjects and boosts blood flow and muscle uptake of glucose. Although not yet explicitly stated, the increased blood flow and lower levels of free fatty acids may be direct effects of the medication or result from lessened glucose toxicity. The improvement in vascular function must be the result of these advantageous effects. Following treatment with 200 mg of metformin, increases in angiogenic (vascular endothelial growth factor), anti-inflammatory (inhibitor kappa B-alpha and interleukin 10), and neurotrophic (myelin basic protein and neural growth factor) factors were more pronounced [24]. As a result, treatment with metformin, particularly at a dose of 200 mg, helped to prevent nerve damage from chronic hyperglycemia [27]. 

In the present study, metformin also increased microvascular circulation by increasing facial nerve blood flow. Acute focal injuries may make the peripheral nerve trunk’s microvascular supply susceptible, especially if they are linked to a direct lesion to the epineurial blood supply. Metformin has antioxidant properties that protect microvascular cells from oxidative damage. Additionally, metformin may prevent structural nerve degeneration, including axonal degeneration from distributed axonal transport, during the diabetic process by preventing microvascular abnormalities through AMPK activation [42]. 

In a rat spinal cord injury model, Wu et al. investigated the function and molecular mechanism of metformin on myelin preservation [43]. They demonstrated that giving metformin (50 mg kg^−1^ d^−1^, ip) to spinal cord injury rats for 28 days greatly enhanced their locomotor function. Additionally, metformin reduced the neuronal apoptosis brought on by spinal cord injury and encouraged axon regrowth. Wu et al. showed that metformin supported microglial cell polarization from M1 to M2, which, in turn, considerably aided the removal of myelin debris and preserved myelin in spinal cord injury rats. By blocking the AMPK-mTOR signaling pathway, metformin also improved the inhibition of autophagic flux brought on by SCI in the spinal cord and the fusion of the autophagosome and lysosome. After damage, metformin increases the expression of LC3-II, which dramatically stimulates autophagy. Furthermore, metformin-induced autophagy decreased the rat lesion site’s cell death. When compared to the control group, it improves the regeneration of nerve tissue, as shown by enhanced expression of the differentiation markers GAP43 and SCG10 and axonal development [44]. In the present study, we compared the effect of metformin on electrophysiological recovery. Currently, the medication metformin is used to treat type 2 diabetes. In addition to the drug’s ability to lower blood sugar, researchers are interested in how it may also affect cancer and cardiovascular disorders. The fundamental mechanisms of action, though, are still unknown. Recently, it has been postulated that the hypoglycemic effects of metformin are caused by metformin-mediated activation of hepatic AMP-activated protein kinase (AMPK). [41]. AMPK is a heterotrimeric enzyme that is expressed in many tissues and plays a central role in the regulation of energy homeostasis.

Wallerian degeneration is the degenerative process that destroys damaged axons and the myelin sheaths that surround them. An essential step in the regeneration process is the elimination of myelin debris. Within two days of injury, the distal stump fragments and Schwann cells begin cleaning the myelin and axonal debris. At the same time, the Schwann cells also proliferate and differentiate. Axonal injury causes the distal fibers to separate from the neuronal stump and experience Wallerian degeneration, in which the neural cytoskeleton breaks down and a lot of axonal and myelin debris is created [45]. The difficulties of nerve regeneration are further exacerbated by the myelin sheet fragments that surround the lesion locations. Therefore, for optimal nerve healing following injury, the velocity and extent of myelin debris clearing are crucial. Serine/threonine (Ser/Thr) kinase member 5′-AMP-activated protein kinase (AMPK) is present in all types of cells and organs. As a cellular energy sensor and regulatory system, AMPK works to maintain the balance between ATP synthesis and consumption in cells [46]. Increased intracellular AMP and ADP levels trigger the activation of AMPK, which facilitates the production of ATP [47]. It is an important endogenous protective molecule that reacts to potentially hazardous stimuli, including diseases like cerebral ischemia, cerebral hemorrhage, and neurodegenerative disorders [48]. Metformin has been shown to have both AMP-activated protein kinase (AMPK)-dependent and AMPK-independent modes of action, including reduction of mitochondrial respiration, inhibition of mitochondrial glycerophosphate dehydrogenase, and a lysosomal mechanism [41]. When normal cells are exposed to stressors known to damage them by producing ROS, metformin has been shown to protect against ROS [49,50]. Metformin’s antineuropathic actions may be caused by activation of AMPK protein kinase 5 (AMPK), and peripheral neuropathy in test animals is associated with poor AMPK signaling. Recently, Nagarajan et al. reported that activation of AMPK can protect against senescence brought on by oxidative stress, both in vivo and in vitro [51]. The limitation of this study is the lack of morphometric studies, which will be further required in the future. 

## 5. Conclusions

Based on our findings, we assume that metformin hastens the recovery of facial nerve crush damage in rats. These findings show that metformin is helpful in promoting nerve regeneration in a rat model of experimental facial nerve crush in the diabetes model. To better understand how metformin affects the facial nerve crush, additional research, including morphological and molecular analysis, is required. 

## Data Availability

Not applicable.
